# The Menstrual Cycle’s Influence on Sleep Duration and Cardiovascular Health: A Comprehensive Review

**DOI:** 10.7759/cureus.47292

**Published:** 2023-10-18

**Authors:** Padigela Rugvedh, Ppavani Gundreddy, Bhushan Wandile

**Affiliations:** 1 Pediatrics, Jawaharlal Nehru Medical College, Datta Meghe Institute of Higher Education and Research, Wardha, IND; 2 Anatomy, Jawaharlal Nehru Medical College, Datta Meghe Institute of Higher Education and Research, Wardha, IND; 3 Medicine, Jawaharlal Nehru Medical College, Datta Meghe Institute of Higher Education and Research, Wardha, IND

**Keywords:** women's health, sleep patterns, hormonal fluctuations, cardiovascular health, sleep duration, menstrual cycle

## Abstract

The menstrual cycle, a fundamental biological process in women, extends its influence beyond reproduction, impacting sleep duration and cardiovascular health. This comprehensive review delves into the intricate connections that bind these three vital aspects of women's health. Beginning with thoroughly exploring the menstrual cycle, we uncover its phases and the dynamic hormonal fluctuations that underlie each stage. We pay special attention to estrogen and progesterone, the primary sex hormones orchestrating the menstrual cycle. With their rhythmic rise and fall, these hormones orchestrate events, affecting sleep duration, sleep patterns, and various indicators of cardiovascular well-being. The review examines how the menstrual cycle influences sleep patterns, exploring the nuanced changes in sleep duration observed throughout menstrual phases. We elucidate the contributing factors, encompassing hormonal fluctuations, the impact of pain and discomfort, and the significance of emotional and psychological factors. All of these elements collectively contribute to variations in sleep quality. Shifting our focus to the cardiovascular system, we investigate the bidirectional relationships between sleep disturbances and cardiovascular conditions, emphasizing the need to address sleep-related issues in the context of cardiovascular risk. The menstrual cycle is analyzed as a pivotal mediator in these intricate connections, exploring how hormonal fluctuations across menstrual phases can influence sleep patterns and cardiovascular health. This analysis provides valuable insights into the complex causality web. As clinical implications emerge, we emphasize the importance of tailoring healthcare strategies for individuals with irregular menstrual cycles. We explore potential interventions, from personalized care and hormone management to lifestyle adjustments, to improve sleep and cardiovascular well-being.

In conclusion, this comprehensive review sheds light on the interplay between the menstrual cycle, sleep duration, and cardiovascular health. It underscores the urgent necessity for personalized healthcare approaches and preventive strategies, empowering women to navigate these intricate relationships. Ultimately, through a nuanced understanding of these interactions, we can work towards enhancing women's overall well-being and reducing cardiovascular risk within the context of menstrual cycle-related influences.

## Introduction and background

The menstrual cycle is a fundamental and intricate physiological process that women experience throughout their reproductive years. It orchestrates a series of hormonal changes and events, driving the monthly release of an egg from the ovaries, known as ovulation, and preparing the body for potential pregnancy. While the menstrual cycle is primarily associated with the female reproductive system, emerging research has revealed its profound and far-reaching influence on women's health, extending well beyond fertility and reproduction [1-3]. These broader effects encompass a range of critical aspects of women's well-being. For instance, it plays a substantial role in bone health, with hormone fluctuations during the menstrual cycle impacting bone density and strength, which is particularly important in osteoporosis prevention [4]. Moreover, the menstrual cycle significantly impacts mental health, with many women experiencing mood changes, irritability, and other emotional variations across different phases of the cycle. Understanding these effects is vital in addressing premenstrual syndrome (PMS) and mood disorders [5]. Additionally, research suggests links between the menstrual cycle and chronic disease risk, including cardiovascular diseases, diabetes, and certain types of cancer. Hormonal fluctuations during the menstrual cycle may influence these risk factors, making it a critical area of study for preventive healthcare [6]. The menstrual cycle is typically divided into distinct phases, each characterized by specific hormonal fluctuations and physiological changes. The cycle begins with the follicular phase, during which the ovaries produce estrogen, stimulating the growth of follicles that contain developing eggs. This phase culminates in ovulation when a mature egg is released from the ovary. Subsequently, the luteal phase ensues, marked by increased levels of progesterone and further changes in the uterine lining in preparation for potential pregnancy. Menstruation commences if conception does not occur, signaling a new cycle's start [1]. Beyond its role in reproduction, the menstrual cycle's hormonal fluctuations exert far-reaching effects on various physiological systems, with significant implications for women's health. For instance, these hormonal changes can impact bone health. During the menstrual cycle, variations in estrogen levels influence bone density, making women more susceptible to conditions like osteoporosis [2]. Additionally, the menstrual cycle can have a profound impact on mental health. Hormonal fluctuations can lead to mood swings, anxiety, and depression, affecting a woman's emotional well-being throughout her cycle [3]. Furthermore, these hormonal shifts may influence chronic disease risk. Research has suggested connections between the menstrual cycle and conditions such as cardiovascular diseases, diabetes, and certain types of cancer. Understanding these links is crucial for preventive healthcare strategies [4]. Sleep and cardiovascular health are two vital aspects of overall well-being. The significance of obtaining sufficient, high-quality sleep is well-established, as it impacts cognitive function, mood, immune function, and metabolic regulation. Similarly, cardiovascular health is paramount, given that cardiovascular diseases remain a leading cause of mortality worldwide [5]. Understanding the factors influencing sleep and cardiovascular health is crucial for developing strategies to promote women's well-being and mitigate the risk of cardiovascular diseases [5]. Recent investigations have begun to shed light on the intriguing relationship between the menstrual cycle and these two critical domains. Researchers have uncovered evidence suggesting that the menstrual cycle may influence sleep duration and quality, potentially contributing to sleep disturbances and disorders experienced by women. Moreover, emerging studies indicate that variations in sex hormone levels across the menstrual cycle may affect cardiovascular health, potentially affecting factors such as blood pressure, lipid metabolism, and inflammation [6]. This comprehensive review aims to explore and synthesize the current body of knowledge regarding the influence of the menstrual cycle on sleep duration and cardiovascular health. We aim to provide a detailed examination of the existing literature, summarizing key findings, identifying gaps in our understanding, and offering insights into the potential mechanisms that underlie these interactions. By elucidating the complex interplay between the menstrual cycle, sleep, and cardiovascular health, we hope to contribute to a more holistic understanding of women's health and provide a foundation for future research in this field.

## Review

Menstrual cycle overview

Phases of the Menstrual Cycle

Menstrual phase: This phase signals the commencement of the menstrual cycle. It is characterized by the shedding of the uterine lining, known as the endometrium, in response to decreasing levels of estrogen and progesterone. This shedding results in menstruation, commonly called a woman's period, typically lasting 3 to 7 days [1].

Follicular phase: Following the menstrual phase, the follicular phase unfolds. During this period, the hypothalamus in the brain releases gonadotropin-releasing hormone (GnRH), which stimulates the pituitary gland to produce follicle-stimulating hormone (FSH). FSH, in turn, kickstarts the development of multiple ovarian follicles, each housing an immature egg or oocyte. Estrogen levels gradually rise throughout the follicular phase. This rise in estrogen encourages the thickening of the endometrial lining and supports the maturation of the dominant follicle that will later release the mature egg [1].

Ovulation: Occurring approximately midway through the menstrual cycle, ovulation is a pivotal event. It is triggered by a luteinizing hormone (LH) surge and a peak in estrogen levels. These hormonal changes stimulate the release of a mature egg from the dominant ovarian follicle. Ovulation typically occurs around the 14th day of a 28-day menstrual cycle and marks the prime time for fertility [3].

Luteal phase: Following ovulation, the luteal phase begins. During this phase, the now-empty follicle transforms into a structure called the corpus luteum. The corpus luteum secretes progesterone along with some estrogen. Progesterone is crucial in preparing the uterine lining for potential embryo implantation and maintaining a pregnancy if conception occurs. If pregnancy doesn't happen, the corpus luteum regresses, leading to a decline in progesterone and estrogen levels. This decline ultimately triggers the start of a new menstrual cycle with menstruation, and the cycle repeats [7].

Hormonal Changes During Each Phase

The menstrual cycle is a highly regulated sequence of hormonal changes where estrogen and progesterone play central roles. Estrogen, specifically estradiol, dominates during the follicular phase of the menstrual cycle. As the ovarian follicles mature, they progressively increase their production of estrogen. This hormone serves various essential functions within the female reproductive system. One of its primary roles is to promote the thickening of the uterine lining, referred to as the endometrium. This thickening is crucial for creating a nourishing environment in case fertilization occurs, supporting potential embryo development. Moreover, estrogen plays a key role in stimulating the LH surge, which initiates ovulation-the pivotal event in the menstrual cycle when a mature egg is released from the ovary. Beyond its reproductive functions, estrogen influences the body, affecting parameters such as bone density, mood regulation, and cardiovascular health, among other physiological processes [8].

Progesterone, on the other hand, takes the spotlight during the luteal phase of the menstrual cycle. It is primarily produced by the corpus luteum, the structure formed from the remains of the ovarian follicle after ovulation. Progesterone's role is multifaceted but pivotal. Its primary function is to maintain the integrity of the endometrial lining, making it conducive to embryo implantation. In the event of conception, progesterone continues its crucial role by providing essential support to the early stages of pregnancy, helping to sustain it until the placenta can take over hormone production. However, if fertilization does not occur, the corpus luteum regresses, leading to a precipitous decline in progesterone levels, which, in turn, signals the onset of menstruation and the initiation of a new menstrual cycle [9].

These hormonal fluctuations are not confined solely to reproductive functions but extend their influence into broader physiological processes. The ebb and flow of estrogen and progesterone orchestrate a delicate interplay within the female body, impacting everything from sleep patterns to cardiovascular health. This nuanced understanding of the hormonal changes during each menstrual cycle phase forms the foundation for comprehending its far-reaching effects on women's well-being [10].

Typical Duration and Variations

The menstrual cycle duration, a hallmark of female reproductive physiology, is not a one-size-fits-all affair. While the average menstrual cycle typically spans around 28 days, it is crucial to recognize that there is a considerable range of normalcy, and cycles can vary widely, falling between 21 to 35 days. These variations arise from many influences, reflecting the intricate interplay of genetics, age, stress, and underlying medical conditions [11].

First and foremost, genetics plays a significant role in determining the length of an individual's menstrual cycle. Family history can often indicate what to expect, as women follow similar patterns to their mothers or sisters. However, variations can occur even within the same family [12]. Age is another influential factor. As hormonal systems mature during adolescence, cycles may be irregular and longer, eventually settling into a more predictable pattern. Conversely, as women approach menopause, cycles often become shorter and more irregular [13].

Stress can profoundly impact the menstrual cycle, whether due to everyday pressures or significant life events. The body's stress response system can disrupt the delicate balance of hormones, potentially leading to missed or irregular periods [14]. Furthermore, it is important to note that certain medications, such as birth control pills and hormonal replacement therapy, can also affect the menstrual cycle. These medications introduce exogenous hormones into the body, altering the natural hormonal fluctuations and sometimes causing changes in cycle length and regularity. It is essential to consider these pharmaceutical factors when examining the menstrual cycle and its various influences. In addition to stress and medication, underlying medical conditions, such as polycystic ovary syndrome (PCOS) or thyroid disorders, can influence cycle length and regularity. These conditions can disrupt the normal hormonal signaling pathways, causing variations in the menstrual cycle [15].

Significantly, not all women experience the same symptoms or hormonal fluctuations during their menstrual cycles. While some may have clockwork-like regularity, others may contend with irregular cycles characterized by unpredictable timing and varying symptoms. Furthermore, the degree of hormonal fluctuations can vary widely, with some women experiencing more pronounced changes in hormone levels than others [16].

Understanding these individual variations is paramount when delving into the potential impact of the menstrual cycle on sleep duration and cardiovascular health. These variations create a rich tapestry of experiences, influenced not only by physiological factors but also by psychological and cultural elements. Individual differences can contribute to diverse outcomes and responses to the menstrual cycle's influence on overall well-being. Recognizing and appreciating this diversity, encompassing physiological, psychological, and cultural aspects, is essential for tailoring healthcare approaches that acknowledge and address each woman's unique needs during her menstrual journey [17].

Sleep duration and quality across the menstrual cycle

Menstrual Cycle and Sleep Patterns

Sleep duration changes: Research has revealed that the menstrual cycle influences sleep duration [18]. Notably, during the luteal phase, which occurs in the latter half of the menstrual cycle following ovulation, some women may experience a reduction in their sleep duration compared to the follicular phase in the first half of the cycle. This sleep duration alteration is closely tied to hormonal changes, particularly the notable increase in progesterone levels during the luteal phase. Progesterone is known to have sedative properties and can contribute to feelings of drowsiness. However, it is crucial to recognize that individual variations exist, and not all women may experience significant changes in their sleep duration due to the menstrual cycle. Age, lifestyle, and overall health can also play a role in these variations, making it a complex and multifaceted relationship [18]. Sleep architecture changes: Beyond sleep duration, the menstrual cycle can also impact sleep architecture, including various sleep stages. Several studies have indicated alterations in rapid eye movement (REM) and non-REM sleep patterns across the menstrual cycle. These shifts in sleep architecture are closely tied to fluctuations in sex hormone levels, primarily estrogen and progesterone. The intricate dance of these hormones throughout the menstrual cycle can affect the proportions and characteristics of different sleep stages, ultimately influencing overall sleep quality. In addition to hormonal influences, lifestyle factors, such as diet, exercise, and caffeine intake, can also play a significant role in sleep patterns during the menstrual cycle. The interplay of these lifestyle factors with hormonal fluctuations can lead to variations in sleep efficiency and subjective sleep satisfaction. This can manifest as feelings of restlessness or disrupted sleep, particularly in women more sensitive to hormonal fluctuations [19].

Factors Influencing Sleep During the Menstrual Cycle

Hormonal fluctuations: One of the primary factors influencing sleep during the menstrual cycle is the dynamic interplay of hormones, specifically estrogen and progesterone. As the menstrual cycle progresses, these hormones undergo significant fluctuations, and these changes can impact sleep patterns during the luteal phase, which follows ovulation, progesterone levels surge. This increase in progesterone is associated with higher body temperature, potentially making it more challenging for some women to fall asleep comfortably. Elevated progesterone levels can also affect neurotransmitters like serotonin and gamma-aminobutyric acid (GABA), essential for regulating sleep. Changes in these neurotransmitter levels can lead to variations in sleep quality, making it more challenging to achieve restorative and uninterrupted sleep. These hormonal shifts highlight the intricate link between the menstrual cycle and sleep, underscoring the need for tailored strategies to address these fluctuations [20].

Pain and discomfort: Menstrual cramps and the general discomfort associated with menstruation can significantly impact sleep quality. Pain and discomfort often lead to difficulty falling asleep and frequent awakenings during the night. These disturbances can result in poorer sleep quality, leaving women feeling less rested upon waking. Therefore, effective management of pain and discomfort during menstruation is crucial for improving sleep. This may involve using over-the-counter pain relievers, heat therapy, or other comfort measures tailored to an individual's needs. By addressing these physical symptoms, women can enhance their sleep experience during their menstrual cycle [21].

Emotional and psychological factors: Emotional and psychological factors, such as stress, anxiety, and mood swings, can fluctuate throughout the menstrual cycle. These emotional variations can significantly contribute to sleep disturbances. Heightened stress or emotional turmoil can interfere with initiating and maintaining sleep, making it challenging to achieve restful and restorative slumber. To mitigate these challenges, cognitive-behavioral strategies and stress-reduction techniques can be beneficial. These may include mindfulness meditation, relaxation exercises, or counseling to help manage emotional and psychological factors that impact sleep. By addressing these aspects of well-being, women can better navigate the emotional ups and downs that can affect their sleep patterns during the menstrual cycle [22].

Sleep Disorders and Menstrual Cycle Interactions

Prevalence of sleep disorders: The interaction between sleep disorders and the menstrual cycle is a notable aspect of women's health. Certain sleep disorders, such as insomnia and restless legs syndrome (RLS), can be more prevalent or worsen during specific phases of the menstrual cycle. Insomnia, characterized by difficulty falling or staying asleep, may become more common during the premenstrual and menstrual phases. It is estimated that 9% to 15% of adults in the United States experience insomnia. Furthermore, RLS, a condition marked by uncomfortable sensations in the legs and an irresistible urge to move them, can worsen during the luteal phase of the menstrual cycle. It is essential to recognize that these interactions can lead to heightened sleep disturbances for women, impacting their overall well-being [23].

Impact on sleep quality and duration: Sleep disorders, when present, can significantly affect the quality and duration of sleep throughout the menstrual cycle. Women who experience sleep disorders may find their symptoms more pronounced during specific phases, amplifying sleep disturbances. These disturbances can encompass difficulties falling asleep, maintaining sleep, or experiencing restorative sleep. The cyclic nature of these sleep disruptions underscores the importance of recognizing and addressing underlying sleep disorders to enhance overall sleep quality and duration. Effective management and treatment of insomnia and RLS can help mitigate the impact of these conditions on women's sleep during the menstrual cycle [21].

Strategies for Improving Sleep During the Menstrual Cycle

Hormonal management: Some women may benefit from hormonal strategies to address hormone-related sleep disturbances. Hormonal contraceptives or prescribed hormonal therapies can help regulate hormonal fluctuations across the menstrual cycle. When appropriately prescribed by healthcare providers, these therapies may assist in stabilizing hormone levels and mitigating sleep disruptions [21].

Pain management: Managing menstrual discomfort and pain is essential for improving sleep during menstruation. Over-the-counter pain relievers like ibuprofen or prescription medications recommended by a healthcare provider can alleviate pain, allowing for more restful sleep [24].

Sleep hygiene: Good sleep hygiene is fundamental to improving sleep quality throughout the menstrual cycle. This includes maintaining a consistent sleep schedule, creating a comfortable sleep environment (e.g., a dark, cool room), and avoiding stimulating activities before bedtime. These habits promote healthy sleep patterns and enhance sleep quality [25].

Stress reduction: Emotional and psychological factors, such as stress and anxiety, can impact sleep. Incorporating stress-reduction techniques into one's daily routine can mitigate the influence of these factors on sleep. Mindfulness meditation, relaxation exercises, or yoga effectively manage stress and promote better sleep [26].

Cognitive-Behavioral therapy (CBT): It is important to note that cognitive-behavioral therapy (CBT) is an effective and evidence-based treatment for insomnia. CBT for insomnia typically involves addressing the underlying thoughts and behaviors contributing to sleep difficulties and teaching strategies to improve sleep quality [27-28].

Consulting a healthcare provider: If sleep disturbances persist or are severe, it is crucial to seek guidance from a healthcare provider or sleep specialist. These professionals can thoroughly evaluate sleep patterns, identify underlying causes, and provide personalized treatment options. They may recommend behavioral therapies, medication, CBT, or other interventions to address specific sleep disorders or menstrual cycle-related sleep challenges [27].

Cardiovascular health and the menstrual cycle

Influence of Hormonal Fluctuations on Cardiovascular Health

Role of estrogen: Estrogen, a pivotal sex hormone with levels that fluctuate throughout the menstrual cycle, profoundly influences cardiovascular health. It is associated with several cardioprotective effects that can impact overall well-being. One of the most notable effects of estrogen is its ability to promote vasodilation, the widening of blood vessels. This vasodilatory effect helps maintain healthy blood pressure levels and ensures efficient blood flow. Estrogen is also recognized for its anti-inflammatory properties, which can contribute to reducing inflammation within the cardiovascular system. Additionally, estrogen tends to improve lipid profiles by increasing high-density lipoprotein (HDL) cholesterol, often referred to as "good" cholesterol, and decreasing low-density lipoprotein (LDL) cholesterol, known as "bad" cholesterol. These combined effects make estrogen a significant player in cardiovascular health. Notably, during the follicular phase of the menstrual cycle, when estrogen levels rise, these cardioprotective effects may be more pronounced, potentially contributing to enhanced cardiovascular well-being [28]. Role of progesterone: Progesterone, another hormone subject to fluctuations during the menstrual cycle, also influences cardiovascular physiology. However, the impact of progesterone on the cardiovascular system appears to be more complex than estrogen's. Progesterone has been associated with specific vascular changes, including increased peripheral resistance. This change in resistance may result in slight blood pressure elevations during the menstrual cycle's luteal phase. While these changes are generally modest, they are noteworthy within the context of cardiovascular health. It is important to emphasize that progesterone's effects on blood pressure are variable and can vary among individuals. Moreover, these changes' significance in overall cardiovascular health is still an area of ongoing research [29].

Effects on Cardiovascular Health Markers Across the Menstrual Cycle

Blood pressure variations: Blood pressure exhibits slight variations across the menstrual cycle, which can be attributed to hormonal fluctuations. Research suggests that blood pressure tends to be lower during the follicular phase, characterized by higher estrogen levels. Conversely, blood pressure may slightly increase during the luteal phase, which coincides with rising progesterone levels. These changes are generally subtle and within the normal range for most women. While these variations may not be clinically significant for the majority, they emphasize the role of hormones in regulating cardiovascular function and the need for individualized monitoring in cases where blood pressure regulation is a concern [30]. Heart rate variability changes: Heart rate variability (HRV), a measure of the variation in time between successive heartbeats, is recognized as an indicator of cardiovascular health. Studies have indicated that HRV can fluctuate across the menstrual cycle. During the follicular phase, when estrogen levels are higher, HRV tends to be elevated. In contrast, during the luteal phase, characterized by rising progesterone levels, HRV may decrease. Reduced HRV has been associated with an increased risk of cardiovascular events, highlighting the potential relevance of these menstrual cycle-related changes in HRV to overall cardiovascular health. However, further research is needed to elucidate these variations' clinical implications fully [31]. Cholesterol level fluctuations: Cholesterol levels, including LDL and HDL cholesterol, may fluctuate during the menstrual cycle. Estrogen, an essential hormone in the menstrual cycle, plays a role in influencing lipid metabolism. During the follicular phase, estrogen's influence may contribute to increased HDL cholesterol levels, often regarded as "good" cholesterol due to its protective effects against cardiovascular disease. However, the overall impact of these hormonal fluctuations on long-term cardiovascular risk is still an area of ongoing research. The relationship between menstrual cycle-related changes in cholesterol levels and the development of cardiovascular disease remains complex and requires further investigation to understand the clinical significance better [32].

Implications for Long-Term Cardiovascular Health

While typically subtle, the menstrual cycle-induced variations in cardiovascular health markers hold significant implications for long-term cardiovascular well-being. These variations prompt essential questions and considerations about potential connections between menstrual cycle characteristics and the risk of cardiovascular disease. Although the research in this area is ongoing and evolving, it has the potential to inform preventive strategies and guide individualized healthcare approaches for women [33]. One crucial aspect of these implications is recognizing that the menstrual cycle may provide a window into a woman's unique cardiovascular profile. Variations in blood pressure, heart rate variability, and cholesterol levels across the menstrual cycle can offer valuable insights into how her cardiovascular system responds to hormonal fluctuations. Understanding these responses may be particularly relevant for women with preexisting cardiovascular risk factors or conditions [34]. Furthermore, research exploring the relationship between menstrual cycle characteristics and cardiovascular disease risk is still nascent. It is essential to continue studying this complex interplay to establish more concrete connections between menstrual cycle-related variations and long-term cardiovascular outcomes. Such investigations could identify specific menstrual cycle patterns or characteristics associated with an increased risk of cardiovascular disease [35]. Ultimately, the evolving understanding of how the menstrual cycle influences cardiovascular health markers underscores the importance of individualized healthcare approaches for women. Recognizing that women's cardiovascular health may be influenced by hormonal fluctuations throughout their menstrual cycle, healthcare providers can tailor preventive strategies and interventions to address their unique needs. By monitoring and assessing cardiovascular health markers in the context of the menstrual cycle, healthcare professionals can work towards optimizing long-term cardiovascular well-being and reducing the risk of heart disease in women [36].

Gender-Specific Considerations in Cardiovascular Health

Recognizing and addressing gender-specific considerations is paramount in the realm of cardiovascular health. Cardiovascular health varies between genders, and research efforts should consider these gender-specific factors. For women, unique cardiovascular risk factors necessitate careful consideration, and understanding how the menstrual cycle interacts with these factors can provide valuable insights into gender-specific cardiovascular health [37]. Unique risk factors for women: Women face a distinct set of cardiovascular risk factors that differ from those typically observed in men. These include risk factors associated with pregnancy, such as gestational diabetes and preeclampsia, as well as those linked to hormonal changes during menopause. These gender-specific factors can have profound implications for a woman's cardiovascular health. For instance, gestational diabetes and preeclampsia during pregnancy can increase the risk of developing cardiovascular disease. Additionally, the hormonal changes accompanying menopause, including a decline in estrogen levels, can impact the cardiovascular system [38]. Interactions with the menstrual cycle: The menstrual cycle represents an additional layer of complexity in understanding gender-specific cardiovascular health. As discussed earlier, hormone fluctuations like estrogen and progesterone across the menstrual cycle can influence cardiovascular health markers. Recognizing these interactions is crucial because they provide insights into how the menstrual cycle may impact a woman's cardiovascular risk profile [39]. Implications for research and healthcare: To advance our understanding of gender-specific cardiovascular health, research should consider the unique factors that affect women. This includes studying how hormonal fluctuations during the menstrual cycle intersect with other risk factors, such as pregnancy-related conditions and menopause. Additionally, healthcare providers should adopt a gender-specific approach when assessing cardiovascular risk in women. This approach should consider traditional risk factors and those specific to women's health experiences [40].

Interactions between sleep, menstrual cycle, and cardiovascular health

Bidirectional Relationships Between Sleep and Cardiovascular Health

Impact of sleep on cardiovascular health: The relationship between sleep and cardiovascular health is bidirectional and intricate. Adequate and restorative sleep is fundamental for maintaining cardiovascular well-being. When sleep is insufficient or of poor quality, it can have significant adverse effects on various risk factors for cardiovascular disease. For instance, chronic sleep deprivation has been associated with an increased risk of developing hypertension (high blood pressure). Poor sleep quality can also contribute to obesity and insulin resistance, risk factors for heart disease. Furthermore, sleep disorders such as obstructive sleep apnea are directly and profoundly linked to cardiovascular conditions. Sleep apnea, characterized by repeated interruptions in breathing during sleep, has been strongly associated with hypertension, arrhythmias (abnormal heart rhythms), and an elevated risk of heart disease. These findings underscore the critical role of sleep in maintaining optimal cardiovascular health [41].

Influence of cardiovascular health on sleep: Conversely, cardiovascular health can exert a significant influence on sleep patterns. Individuals with various cardiovascular conditions may experience sleep disturbances due to underlying health issues. For instance, heart failure, a condition in which the heart cannot pump blood effectively, can lead to nocturnal dyspnea, characterized by shortness of breath at night. This symptom can disrupt sleep and result in frequent awakenings. Similarly, hypertension can contribute to nighttime awakenings and sleep fragmentation, further impairing sleep quality. Conditions like coronary artery disease may also cause discomfort or pain, leading to sleep disturbances. The intricate interplay between cardiovascular health and sleep underscores the importance of addressing both aspects in the context of overall well-being [42].

Menstrual Cycle as a Mediator

How the menstrual cycle might modulate these interactions: The menstrual cycle is a crucial mediator in the bidirectional relationship between sleep and cardiovascular health. Variations in hormone levels throughout the menstrual cycle can profoundly affect sleep patterns and cardiovascular physiology. Here is how the menstrual cycle might modulate these interactions [6].

Hormonal fluctuations and sleep: The menstrual cycle is marked by significant hormonal fluctuations, with estrogen and progesterone playing central roles. During the luteal phase, which follows ovulation, progesterone levels rise notably. This surge in progesterone can lead to an increase in body temperature, which may affect sleep quality. Elevated body temperature can interfere with the ability to fall asleep comfortably and maintain restful slumber, especially if the sleep environment is not adequately conducive to temperature regulation [3].

Hormonal regulation of cardiovascular factors: The menstrual cycle's hormonal changes can also influence cardiovascular factors. For instance, hormones like estrogen and progesterone are known to impact blood pressure regulation. Estrogen has vasodilatory properties, helping to maintain healthy blood pressure levels. In contrast, progesterone's effects on blood pressure regulation are more complex and may result in slight elevations in blood pressure during the luteal phase. These hormonal changes can also affect inflammatory processes within the body, with estrogen exhibiting anti-inflammatory properties. Inflammation is a key player in cardiovascular health, as chronic inflammation is associated with atherosclerosis and other heart-related conditions. The menstrual cycle's influence on inflammation affects long-term cardiovascular well-being [43].

Clinical implications

Managing Sleep and Cardiovascular Health for Individuals With Irregular Cycles

Individuals with irregular menstrual cycles may encounter unique challenges when managing sleep and cardiovascular health, whether due to hormonal imbalances, conditions like polycystic ovary syndrome (PCOS), or other factors. Healthcare providers should be attentive to these variations when assessing cardiovascular risk and offering sleep-related recommendations. It is essential to recognize that irregular cycles can disrupt the delicate hormonal balance that influences sleep and cardiovascular physiology. As such, tailored approaches to healthcare are crucial in addressing the specific needs and concerns of individuals with irregular cycles [44].

Potential Interventions

Personalized care: It is essential to tailor healthcare strategies to individuals based on their unique menstrual cycle characteristics, sleep patterns, and cardiovascular risk factors. Recognizing that every person's experience is distinct allows healthcare providers to provide more effective and targeted interventions. This approach can involve personalized recommendations for sleep and cardiovascular health management [45]. Sleep assessment: Conducting thorough sleep assessments is fundamental in addressing sleep-related concerns. These assessments should encompass various aspects of sleep, including sleep quality, duration, and the presence of any sleep disorders. Identifying specific sleep-related issues is crucial for developing appropriate interventions and treatment plans [46]. Hormonal management: For individuals with irregular menstrual cycles or hormonal imbalances contributing to sleep disturbances, hormonal management may be considered under the guidance of healthcare providers. Hormone therapy or contraceptives can help regulate hormonal fluctuations, improving sleep quality and overall well-being. The choice of hormonal intervention should be made individually, considering the underlying hormonal imbalances and the person's health goals [47]. Lifestyle modifications: Promoting healthy sleep hygiene and lifestyle modifications is integral to enhancing sleep and cardiovascular health. Encouraging individuals to establish and maintain a regular sleep schedule, create a comfortable sleep environment, and adopt relaxation techniques can significantly benefit sleep quality. Additionally, emphasizing regular physical activity, a balanced diet, and effective stress management can positively impact well-being [25]. Screening for sleep disorders: Identifying and treating sleep disorders is critical to managing cardiovascular health. Healthcare providers should be vigilant in screening for conditions such as sleep apnea, which can exacerbate cardiovascular issues. Timely diagnosis and treatment of sleep disorders, including interventions such as continuous positive airway pressure (CPAP) therapy for sleep apnea, can significantly improve sleep quality and cardiovascular outcomes [48].

Future research directions

Longitudinal Exploration of Menstrual Cycle Variability

Future studies should prioritize long-term research involving large and diverse cohorts to comprehensively assess the impact of menstrual cycle variability on sleep and cardiovascular health. Longitudinal data collection will allow researchers to account for individual differences, hormonal fluctuations, and lifestyle changes over time. This approach will better understand how sleep patterns and cardiovascular health evolve in the menstrual cycle [49].

Integration of Advanced Hormone Profiling

Advancements in hormone profiling technologies, such as continuous monitoring of hormone levels, offer an opportunity to obtain more accurate and detailed data on hormonal fluctuations. Integrating these advanced methods into research can provide a deeper insight into how specific hormones, their fluctuations, and their interactions contribute to sleep disturbances and cardiovascular risk across the menstrual cycle [50].

Diversity and Inclusivity in Research

Future studies should prioritize inclusivity and aim to include diverse populations, including individuals from various ethnic, cultural, and socioeconomic backgrounds. This approach will facilitate a more comprehensive understanding of how menstrual cycle-related influences on sleep and cardiovascular health may vary across different demographic groups, ensuring that research findings apply to a broader population [51].

Objective Sleep Monitoring Techniques

While self-reported data provide valuable insights, incorporating objective sleep monitoring techniques like polysomnography and actigraphy can enhance the precision and reliability of sleep data collection. These methods offer a more detailed assessment of sleep architecture and quality, allowing a better understanding of how sleep patterns change across the menstrual cycle [52].

Interventional Studies and Personalized Medicine

Conducting interventional studies that target sleep and hormonal regulation in individuals with menstrual cycle-related sleep disturbances or cardiovascular risk factors is crucial. These studies can help identify effective interventions tailored to individual needs, such as hormone therapies, lifestyle modifications, or sleep interventions. Personalized medicine approaches can optimize sleep management and cardiovascular health during the menstrual cycle [53].

Utilization of Big Data and Machine Learning

Leveraging big data analytics and machine learning techniques to analyze extensive datasets holds the potential to uncover intricate patterns and associations. By mining large-scale data sources, researchers can identify subtle interactions, predict sleep and cardiovascular outcomes, and refine models for assessing the menstrual cycle's influence on health [54].

Incorporating these future research directions into studying the menstrual cycle's impact on sleep duration and cardiovascular health will deepen our understanding and offer practical insights to inform healthcare practices, preventive measures, and personalized interventions for women's health. This research will enhance individuals' overall well-being and cardiovascular outcomes across diverse menstrual cycle profiles [55].

## Conclusions

In conclusion, this comprehensive review has shed light on the intricate and often underappreciated connections between the menstrual cycle, sleep duration, and cardiovascular health. By meticulously examining the menstrual cycle's phases and hormonal dynamics, we have unraveled its multifaceted impact on women's well-being. From variations in sleep duration and architecture to subtle changes in cardiovascular markers, the menstrual cycle plays a vital role beyond reproduction. Moreover, we have underscored the bidirectional nature of interactions, emphasizing the importance of addressing sleep disturbances and cardiovascular risk in a holistic approach to women's health. The implications of these findings are profound, calling for personalized healthcare strategies that consider individual variations in the menstrual cycle. This review also issues a call to action for further research, advocating for longitudinal studies, advanced profiling techniques, and increased awareness among healthcare providers and policymakers. By recognizing and understanding these complex relationships, we can pave the way for improved women's health, tailored interventions, and, ultimately, reduced cardiovascular risk across diverse menstrual cycle profiles.
